# Substitution of Cannabis for Prescribed Medications in the Management of Anxiety and Depression Among Midlife Women: A Cross‐Sectional Study

**DOI:** 10.1002/hsr2.72746

**Published:** 2026-06-28

**Authors:** Jocelyn Mueller, Jamia Sapp, Jennifer Attonito, Aditya Chakraborty, Karina Villalba

**Affiliations:** ^1^ Department of Psychiatry University of California San Diego California USA; ^2^ Population Health Sciences University of Central Florida Orlando Florida USA; ^3^ Health Administration Programs Florida Atlantic University Boca Raton Florida USA; ^4^ Department of Epidemiology, Biostatistics, and Environmental Health Old Dominion University Norfolk Virginia USA

**Keywords:** anxiety, cannabis use, depression, prescription medication substitution, self‐medication, women's mental health

## Abstract

**Background and Aims:**

Given the rising prevalence of cannabis use as an alternative strategy for managing mental health symptoms, there is a crucial need to understand the determinants of substitution behavior, particularly among midlife women who may experience distinct psychosocial and clinical profiles. This study examined the predictors of cannabis substitution among midlife women by comparing profiles of women who substituted cannabis for antidepressant medications versus those who substituted for anxiolytic medications.

**Methods:**

A cross‐sectional survey of 172 women who reported substituting cannabis for either anxiety or depression medications was conducted. Measures included the PHQ‐8, OASIS, AUDIT, and ACEs. Logistic regression was used to identify predictors of substitution behavior.

**Results:**

Among participants, 88 (51%) substituted cannabis for antidepressants and 84 (49%) for anxiolytics. Clinically significant anxiety (*M* = 12.5, SD = 4.3) and depression (*M* = 11.6, SD = 5.3) were observed. Average ACE scores exceeded four in both groups. In multivariate models, sleep difficulties (OR = 4.2, 95% CI: 2.1–8.0) and alcohol use (OR = 1.1, 95% CI: 1.01–1.09) predicted substitution for anxiety. For depression, predictors included sleep difficulties (OR = 6.6, 95% CI: 3.1–14.1), alcohol use (OR = 1.1, 95% CI: 1.1–1.2), lower education (OR = 0.71, 95% CI: 0.53–0.95), and loneliness (OR = 2.0, 95% CI: 1.2–3.8).

**Conclusion:**

Sleep difficulties and alcohol use were found to be strong predictors for substituting cannabis for anxiety and depression medications among middle‐aged women. These findings highlight the need for clinical awareness of symptom‐specific substitution patterns and emphasize the importance of addressing co‐occurring sleep and substance use problems.

## 1 Introduction

Depression and anxiety are highly prevalent mental health conditions, particularly among women, who are approximately twice as likely as men to experience these conditions [1, 2]. Women using prescription medications such as antidepressants and anxiolytics report several side effects, such as agitation, anxiety, sleeping disorders, headaches, and weight gain [3]. These issues have prompted increasing interest in non‐pharmaceutical alternatives, including cannabis [4]. Although cannabis is not currently approved by the FDA for the treatment of anxiety or depression, a growing body of research has examined its potential to reduce anxiety and depression symptoms [5], with research consistently showing that anxiety and depression are among the most common reasons individuals turn to cannabis [6, 7, 8]. In contrast, recent comprehensive reviews conclude that evidence remains insufficient to recommend cannabinoids as treatments for anxiety or depression [9]. Additionally, it has been found that the belief in the therapeutic benefits of cannabis, whether real or perceived, can potentially affect anxiety and depression symptoms [10]. Women are prescribed anxiolytics and antidepressants at higher rates than men [11, 12] and report higher rates of adverse effects and dissatisfaction with prescribed medications [13]. Despite the prevalence of depression and anxiety, women remain underrepresented in cannabis research, and sex‐specific findings are infrequently reported.

Previous research shows that individuals who are more likely to use substances to cope, are less likely to drink on days they also used cannabis, suggesting those who are heavy drinkers and use cannabis may be particularly prone to substitution behaviors [14]. Insomnia and sleep problems, which are associated with anxiety and depression [15], are among the most commonly cited reasons for medical cannabis use, and potentially for substitution behaviors. Cannabis has been reported as highly effective in assisting with sleep, with many reducing their use of traditional sleep medications as a result [16]. Fatigue, another symptom of anxiety and depression [17], has been reported by women as a reason for using cannabis [7], with findings reporting positive effects on energy and daytime function [18]. Loneliness has been previously found as a predictor for cannabis use via elevated psychological distress, suggesting that cannabis use may function as a coping response to loneliness‐driven distress rather than physical health concerns [19]. Irritability has been documented as a symptom of both anxiety and depression and may be a predictor of substitution behaviors [20]. These pathways may create the perception of treating anxiety or depression and reinforcing continued use as a form of self‐medication.

Few studies have focused on cannabis substitution [4, 7, 21], and none have specifically focused on women with anxiety and depression symptoms. Existing literature has examined cannabis substitution broadly, focusing on general medication use rather than identifying substitution patterns among medication classes [22]. A study found that cannabis users perceived a 50% decrease in depression and 58% in anxiety symptoms [7]. Similarly, Rosenthal and Pipitone [23] found that the majority of medical cannabis patients in Florida reported reducing or discontinuing at least one prescription medication, with anxiety among the most commonly cited reasons for initiating use. A qualitative study explored reasons for transitioning to cannabis, with the majority reporting that cannabis reduces adverse side effects, and many reducing or discontinuing their prescription medication [24]. Another study showed that substitution of cannabis included the potential to improve quality of life and reduce the risk of misuse compared to prescription medications [21]. Antidepressants are the most commonly substituted medications [4], but Piper et al. [25] found that among medical cannabis patients, approximately two‐thirds reported reducing anti‐anxiety and sleep medication use following the initiation of cannabis. Studies have identified that 46%–83% of those taking antidepressants had suboptimal adherence, either skipping doses, not taking the medication as directed, or discontinuing the medication prematurely.

Emerging evidence suggests that some women view cannabis as a perceived alternative to prescription medications, particularly for managing symptoms of anxiety and depression. Although this area of research is still developing, early findings suggest that cannabis may function as a substitution behavior for some individuals with mental health conditions, even in the absence of clinical guidelines supporting its use [21, 26]. Given the growing interest in the therapeutic potential of cannabis, it is critical to better understand its effects on mental health outcomes and how individuals are incorporating it into their treatment decisions.

This study aimed to identify the demographic and clinical predictors of cannabis substitution in midlife women and compared these predictors across two medication classes: anti‐anxiety and antidepressant medications.

## 2 Methods

### 2.1 Study Sample

This online cross‐sectional study assessed a cohort of 412 women 18 years or older, who reported cannabis use [tetrahydrocannabinol (THC) and cannabidiol (CBD)] within the past 12 months. Recruitment for the study was done through a virtual platform from 2022 to 2023 using Qualtrics, an online survey platform that maintains a database of pre‐registered volunteer participants who have opted in to participate in research. Qualtrics distributed the survey electronically to members who met the study's eligibility criteria. This study was approved by the Institutional Review Board at the University of Central Florida (Protocol #: IRB00001138, IRB00012110). Informed consent was obtained from all participants being included in the study.

### 2.2 Measures

#### 2.2.1 AUDIT

Alcohol Use Disorders Identification Test (AUDIT; [27]) is a screening tool developed by the World Health Organization to assess participants' alcohol consumption and alcohol‐related problems. It consists of 10 questions that measure the amount and frequency of alcohol intake, signs of dependence, and harmful consequences of drinking. The total score ranges from 0 to 40, with higher scores indicating a greater likelihood of harmful drinking behavior or alcohol dependence: 0–7 (low risk), 8–15 (moderate risk), 16–19 (high risk), and 20 or more (possible dependence).

#### 2.2.2 ACES

The Adverse Childhood Experiences (ACES; [28]) measure is a tool used to assess the cumulative impact of various forms of childhood trauma and adversity. It includes 10 questions on experiences such as abuse (physical, emotional, or sexual), neglect, and household dysfunction (e.g., parental mental illness, substance abuse, domestic violence, or incarceration). A score of 0 indicates no adverse experiences, while a score of 1–3 suggests low to moderate exposure to adversity. A score of 4 or higher indicates high exposure to adversity.

#### 2.2.3 PHQ‐8

The Patient Health Questionnaire depression scale (PHQ‐8; [29]) is a tool used to measure current depression. It includes eight items that evaluate the frequency of depressive symptoms—such as feeling down, loss of interest or pleasure, sleep issues, and changes in appetite or energy levels—over the past 2 weeks, using a scale from 0 (not at all) to 3 (nearly every day). The total score ranges from 0 to 24, with higher scores indicating more severe depressive symptoms. A score of 10 or higher is commonly used to identify current depression.

#### 2.2.4 OASIS

The Overall Anxiety Severity and Impairment Scale (OASIS; [30]) asks a series of questions regarding their anxiety symptoms and how it affects their daily life. The scale consists of five questions that assess the severity and impairment associated with anxiety. Each item is rated on a 5‐point Likert scale ranging from 0 (none) to 4 (extreme). The total score, ranging from 0 to 25, provides an overall assessment of anxiety severity and its impact on functioning. Total scores of 5 or less represent no to mild anxiety, between 6 and 10 represent moderate anxiety, between 11 and 15 represent severe anxiety, and 16 or higher represent extreme anxiety.

#### 2.2.5 Other Predictors

Individual binary questions were asked to assess key predictors in this study, including whether respondents experienced difficulty with sleep (e.g., trouble falling or staying asleep, frequent napping) and if they struggled to cope with stress more than usual (Yes/No). Participants were also asked if they used cannabis to address these issues. Additional questions inquired about feelings of loneliness, irritability, and fatigue (Yes/No). See Table 2.

#### 2.2.6 Outcome Measure

The outcome measure was assessed by the following question, “Did you substitute cannabis for anti‐depressants?” (Yes/no) “Did you substitute cannabis for anti‐anxiety prescription drugs?” (Yes/No). These questions were examined as separate outcome measures.

### 2.3 Statistical Analysis

Descriptive statistics summarized the demographic and clinical characteristics of the study sample. Continuous variables, such as age and depression, were reported as means and standard deviations for normally distributed data. Categorical variables, including education level, employment status, and health status, were presented as percentages.

For comparative analyses between women who used cannabis as a substitute for prescription medications for anxiety versus those who used it for depression, *χ* ^2^ tests were employed to assess differences in categorical variables, while independent samples *t*‐tests were applied to continuous variables. The normality of continuous variables was evaluated using the Shapiro–Wilk test.

A logistic regression model using the Backward Likelihood Ratio was constructed to evaluate the associations between demographic and clinical factors (e.g., age, education, alcohol use) and the likelihood of substituting cannabis for prescription medications. Variables significant in bivariate analyses or deemed clinically relevant were included in the multivariate logistic regression model. Adjusted odds ratios (ORs) with 95% confidence intervals (CIs) were calculated to estimate the strength of associations. Statistical significance was determined using a two‐tailed *p* value of < 0.05 for all analyses. All statistical analyses were conducted using IBM SPSS Statistics (Version 27; IBM Corp). Statistical significance was evaluated using a prespecified two‐sided *α* level of 0.05 for all tests.

## 3 Results

### 3.1 Demographic Characteristics

Notably, 172 of the 412 women sampled (41.7%) reported substituting prescription medications with cannabis for the management of anxiety or depression, indicating that cannabis substitution was prevalent behavior in the midlife sample. Among them, 88 (51%) substituted cannabis for antidepressants, while 84 (49%) substituted it for anxiolytic medications. The mean age was 52.2 years (SD = 11.4) among those treating anxiety and 55.3 years (SD = 12.8) among those treating depression. The majority of participants identified as White 70 (83%) for those replacing anxiety medications and 70 (80%) for those replacing antidepressants. Black women accounted for 12 (14%) for those replacing anxiety and 15 (18%) replacing antidepressants, while Hispanic women represented 10 (33%) and 10 (11%), respectively. Marital status and educational attainment were comparable across both groups. Approximately half of the participants were employed, 40 (47%) for those replacing anxiety medications and 41 (48%) for those replacing antidepressants. No statistically significant demographic differences were observed between women who substituted cannabis for anxiety versus depression. See Table 1.

**Table 1:** Demographic characteristics among women who reported substituting cannabis for anxiety and depression medication.

Characteristics	Anxiety medication (*n* = 84) *n*(%)	Depression medication (*n* = 88) *n*(%)	*p*
Age, mean (SD)	52.2 (11.4)	55.3 (12.8)	0.04
Race			0.09
White	70/84 (83%)	70/88 (80%)	
Black	12/84 (14%)	15/88 (17%)	
Other	2/84 (5%)	2/88 (2%)	
Hispanic			0.68
Yes	10/84 (33%)	10/88 (11%)	
No	74/84 (66%)	78/88 (89%)	
Marital status, No. (%)			0.57
Single	9/84 (11%)	9/88 (10%)	
Married	30/84 (35%)	28/88 (32%)	
Separated	7/84 (8%)	12/88 (14%)	
Divorced	20/84 (24%)	24/88 (27%)	
Widowed	5/84 (6%)	4/88 (4%)	
Living with a partner	13/84 (15%)	11/88 (12%)	
Education, No. (%)			0.22
Some high school or less	8/84 (8%)	6/88 (7%)	
High school diploma	15/84 (18%)	21/88 (24%)	
Some college	32/84 (38%)	30/88 (34%)	
College or trade school	21/84 (25%)	22/88 (25%)	
College graduate	11/84 (13%)	9/88 (10%)	
Employed No. (%)			0.28
Yes	40/84 (47%)	41/88 (48%)	
No	44/84 (52%)	47/88 (43%)	
Self‐health rate			0.08
Very good	16/84 (19%)	20/88 (23%)	
Good	32/84 (38%)	32/88 (36%)	
Fair	25/84 (30%)	25/88 (28%)	
Poor	11/84 (13%)	11/88 (12%)	
Very poor	0/84 (0%)	0/88 (0%)	

*Note: χ* ^2^ tests were used for categorical variables. Statistical significance was evaluated at *p *< 0.05 (two‐tailed).

### 3.2 Clinical Characteristics

Women who reported substituting cannabis for prescription medications to manage anxiety and depression reported mean OASIS scores of 12.5 (SD = 4.3) for the anxiety group and 12.5 (SD = 3.9) for the depression group, both exceeding the established cut‐off point of 10 for clinically significant anxiety. The PHQ‐8 range indicates 10–14 as moderate depression symptoms, with mean scores in our sample of 11.3 (SD = 5.7) for the anxiety group and 11.9 (SD = 4.9) for the depression group. Alcohol use did not reach the threshold for hazardous consumption in either group. Adverse Childhood Experiences (ACEs) scores averaged 4.0 (SD = 2.9) among those substituting for anxiety and 4.1 (SD = 2.7) among those substituting for depression. Significant differences were observed in symptom presentation between groups. Women who substituted cannabis for prescription medications to manage depression reported higher rates of sleep difficulties, 55 irritability 55 (62%), and stress 55 (63%) compared to those who substituted for anxiety (sleep difficulties (45/54%, 44/53%, 44/53%, respectively) 44 (53%) to 44 (53%)). However, no significant differences were observed. See Table 2.

**Table 2:** Clinical characteristics among women who reported substituting cannabis for anxiety and depression medication.

Clinical characteristics	Anxiety medication (*n* = 84) *n*(%)	Depression medication (*n* = 88) *n*(%)	*p*
Depression, score mean (SD)	11.3 (5.7)	11.9 (4.9)	0.90
Anxiety, score mean (SD)	12.1 (4.3)	12.5 (3.9)	0.12
AUDIT, score mean (SD)	6.5 (8.3)	6.8 (8.1)	0.87
ACES, score mean (SD)	4.0 (2.9)	4.1 (2.7)	0.97
Difficulties with sleep No. (%)			0.24
Yes	45/84 (54%)	55/88 (63%)	
No	39/84 (46%)	31/88 (37%)	
Loneliness No. (%)			0.45
Yes	40/84 (47%)	42/88 (48%)	
No	43/84 (53%)	46/88 (59%)	
Irritability No. (%)			0.21
Yes	42/84 (52%)	55/88 (62%)	
No	39/84 (46%)	32/88 (37%)	
Stress No. (%)			
Yes	44/84 (53%)	55/88 (63%)	0.18
No	40/84 (45%)	33/88 (37%)	
Fatigue No. (%)			
Yes	41/84 (49%)	45/88 (51%)	0.76
No	43/84 (50%)	43/88 (49%)	

*Note:* Independent samples *t*‐tests were used for continuous variables (age, depression, anxiety, AUDIT, ACES scores).

### 3.3 Logistic Models

We conducted separate bivariate logistic regression analyses for anxiety and depression to assess the associations between demographic and clinical variables and the likelihood of substituting cannabis for prescription medications among women using cannabis to manage these conditions.

### 3.4 Anxiety Model

In the bivariate analysis, significant factors associated with substituting cannabis for prescription medications to manage anxiety included lower income, sleep difficulties (OR = 2.7, 95% CI: 1.6–4.2), irritability (OR = 2.4, 95% CI: 1.5–4.1), fatigue (OR = 2.9, 95% CI: 1.4–4.6), stress (OR = 2.5, 95% CI: 2.4–4.7), alcohol use (OR = 1.1, 95% CI: 1.02–1.10), and adverse childhood experiences (OR = 1.2, 95% CI: 1.05–1.24). In the multivariate model, which included all variables that were significant in the bivariate analysis, only alcohol use (OR = 1.1, 95% CI: 1.01–1.09) and sleep difficulties (OR = 4.2, 95% CI: 2.1–8.0) remained statistically significant predictors of prescription medication substitution.

### 3.5 Depression Model

In the bivariate analysis for substitution of antidepressant medications with cannabis, significant predictors included younger age (OR = 0.97, 95% CI: 0.96–0.99), lower educational attainment (OR = 0.77, 95% CI: 0.66–0.97), sleep difficulties (OR = 3.2, 95% CI: 1.9–5.4), loneliness (OR = 2.8, 95% CI: 1.7–4.7), fatigue (OR = 2.6, 95% CI: 1.5–4.4), irritability (OR = 3.9, 95% CI: 2.3–6.7), alcohol use (OR = 1.1, 95% CI: 1.03–1.11), and adverse childhood experiences (OR = 1.2, 95% CI: 1.06–1.26). Variables significant at the bivariate level were subsequently entered into a multivariate logistic regression model using the Backward Likelihood Ratio method. In the final model, lower education (OR = 0.71, 95% CI: 0.53–0.95), alcohol use (OR = 1.1, 95% CI: 1.1–1.2), sleep difficulties (OR = 6.6, 95% CI: 3.1–14.1), and loneliness (OR = 2.0, 95% CI: 1.2–3.8) remained significant independent predictors of cannabis substitution for antidepressant medications (see Table 3).

**Table 3:** Multivariate analysis among women who reported substituting cannabis for anxiety and depression prescription medication.

	*β*	SE	Wald	*p*	OR	95% CI
**Cannabis substitution for anti‐anxiety medication (*n* = 84)**						
AUDIT	0.045	0.022	4.17	0.041	1.1	1.01–1.09
Difficulties with sleep (yes/no)	1.44	0.332	18.8	0.001	4.2	2.1–8.0
**Cannabis substitution for anti‐depression medication (*n*** = **88)**						
Education	−0.334	0.023	5.01	0.02	0.71	0.53–0.95
AUDIT	0.054	0.384	5.56	0.001	1.1	1.1–1.2
Difficulties with sleep (yes/no)	1.89	0.310	24.2	0.05	6.6	3.1–14.1
Loneliness (yes/no)	0.609	0.692	6.98	0.008	2.0	1.2–3.8

## 4 Discussion

This study examined whether demographic and clinical predictors of cannabis substitution differed between antidepressants and anxiolytics among middle‐aged women experiencing symptoms of anxiety and depression. This comparison is supported as antidepressants and anxiolytics carry distinct side‐effect profiles and elicit different patient perceptions, both of which may differentially shape motivations for substitution and the clinical symptom profiles associated with it [21]. Among the key findings, sleep difficulties and alcohol use emerged as significant predictors. Women reporting sleep difficulties were six times more likely to substitute cannabis for anxiolytics and four times more likely to substitute cannabis for antidepressants. These results suggest that sleep impairment was associated with a higher likelihood of cannabis substitution.

Sleep difficulties have emerged as one of the most commonly cited reasons for using cannabis. Several studies have demonstrated that patients report improved sleep quality, reduced reliance on conventional psychiatric drugs, and fewer side effects after initiating cannabis use [21, 25]. More recent evidence supports these findings, identifying sleep as a leading motive for cannabis substitution, especially among users seeking alternatives to prescription drugs perceived as less effective or poorly tolerated [4]. However, recent systematic reviews have failed to find sufficient evidence supporting cannabinoid use for sleep [9]. Our findings highlight that sleep difficulties are not only prevalent in this demographic but also associated with a higher likelihood of cannabis substitution. Nonetheless, the therapeutic implications of cannabis use depend substantially on its chemical composition. Evidence indicates that cannabis products with high THC content may provide short‐term relief from insomnia and sleep‐onset difficulties [31]. Yet, these same products have been associated with disruptions in rapid eye movement (REM) sleep, a stage crucial for emotional and cognitive regulation, and have been associated with changes in sleep architecture over time [32, 33]. In contrast, CBD‐dominant formulations have shown promise in stabilizing sleep patterns and reducing anxiety‐induced arousal, suggesting a potentially safer and more sustainable approach to addressing both sleep and psychiatric symptoms simultaneously [34, 35]. Importantly, while individuals may be motivated to substitute cannabis for prescription medications, cannabis use carries its own clinically significant risk profile, including cannabis use disorder, psychosis risk, and cardiovascular effects [36, 37, 38]. These risks must be weighed against any perceived benefits, particularly in the context of self‐directed or unsupervised use. An alternative to cannabis substitution is Cognitive Behavior Therapy for Insomnia (CBT‐I), which remains the first‐line recommended treatment for sleep disorders. Future research should examine whether individuals who substitute cannabis for prescription medications are aware of these risks and whether they have been offered or tried evidence‐based alternatives.

The relationship between alcohol as a predictor for cannabis substitution for prescribed medications among middle‐aged women with anxiety and depression symptoms is a critical yet understudied area of research. In this study, we identified alcohol use as a predictor of cannabis substitution for prescription medication. Although cannabis substitution may reduce reliance on prescription medications for some individuals, emerging evidence suggests that its role in substance‐use behaviors is complex. While some individuals substitute cannabis to reduce medication dependence, others may engage in polysubstance use, where cannabis consumption is associated with increased alcohol intake. For example, Galaj and Xi [39] found that in certain populations, cannabis use can reinforce alcohol consumption patterns, potentially complicating efforts to reduce overall substance use. These findings underscore the importance of considering individual differences in substance‐use behaviors and motivations for substitution. Moreover, the observed average ACEs scores above 4 suggest high levels of early‐life trauma, a known risk factor for both psychiatric morbidity and substance use, which may further explain the appeal of cannabis as a self‐directed coping mechanism [28, 40].

The findings from this study have several important implications for clinical practice and public health. First, the substitution of cannabis for prescription medications highlights the need for healthcare providers to engage in open, informed discussions with patients regarding the potential risks and benefits of cannabis use. Second, it is essential to address sleep difficulties as part of mental health care, providing evidence‐based treatments for insomnia and other sleep‐related disorders. Furthermore, research is needed to examine optimal dosing and the specific components of cannabis products, such as THC and CBD ratios, to understand their distinct effects on mental health and guide safe therapeutic use. Future research should prioritize longitudinal studies to evaluate the long‐term effects of substituting cannabis for prescription medications on mental health outcomes [41].

### 4.1 Limitations and Future Research Dimensions

While this study provides important insights into the factors associated with cannabis substitution for anxiety and depression medications, several limitations should be noted. First, the cross‐sectional design limits our ability to infer causality. It is unclear whether cannabis use led to worse mental health outcomes or whether women with more severe symptoms were more likely to substitute cannabis for their prescriptions. Longitudinal studies are needed to disentangle these relationships and determine the long‐term effects of cannabis substitution. These findings reflect self‐reported symptoms and substitution behaviors and should not be interpreted as evidence of clinical efficacy. Cannabis is not approved by the FDA for the treatment of sleep disorders, anxiety, or depression. Another important limitation is that the substitution outcome was measured using two single yes/no items asking whether participants had substituted cannabis for antidepressants or anti‐anxiety prescription drugs. These items did not assess duration, chronicity, or whether cannabis was used concurrently with ongoing prescription medications. Consequently, our findings should be interpreted as reflecting any self‐reported history of substitution rather than sustained substitution patterns, and we cannot draw conclusions about whether cannabis or prescribed medications had a greater impact on participants' symptoms. Prospective studies with more detailed measures of substitution frequency, duration, and temporality are needed to determine how different substitution patterns influence clinical outcomes. The current study did not assess the specific anxiolytic or antidepressant medications reported by participants, nor the specific medications for which cannabis was being substituted. Since many medications can be used for both anxiety and depression, this limits our ability to draw conclusions about substitution patterns at the level of specific medications. The results of this study should be interpreted as medication‐class‐level rather than drug‐specific. Researchers are encouraged to collect more detailed pharmacological assessments in future work. Furthermore, we did not assess the temporal order in which participants substituted cannabis for their prescription medications, leaving it unclear whether they initially used traditional medications and later transitioned to cannabis or chose cannabis from the outset instead of starting antidepressants or anxiolytics. The inclusion criteria for participants to have a history of cannabis use may have influenced substitution behaviors and limited the generalizability of our findings to broader populations. Addressing these limitations in future research would provide greater clarity on prescription medication behaviors and cannabis substitution. Future research should also compare cannabis substitution and non‐substitution samples to determine whether the predictors identified in the present study are specific to substitution behavior or reflect broader correlates of cannabis use among midlife women with anxiety or depression. Additionally, the reliance on self‐reported data introduces the possibility of recall bias or underreporting, particularly regarding substance use and mental health symptoms. Furthermore, the study population consisted primarily of women over 50, which may limit the generalizability of the findings to other age groups or genders. Finally, midlife women who substituted cannabis for prescription medications may represent a self‐selecting group, as they were more likely to have been prescribed medications for anxiety or depression. This prescription history may have facilitated the opportunity to replace these treatments with cannabis, highlighting a potential selection bias in the study.

## 5 Conclusion

This study emphasizes the increasing trend of cannabis substitution for prescription medications among women experiencing moderate levels of anxiety and depression. Sleep difficulties and alcohol use emerged as significant predictors of cannabis substitution, emphasizing the multifactorial nature of this behavior. These findings underscore the need for further research to clarify the relationship between cannabis use, mental health outcomes, and co‐occurring substance use. A deeper understanding of these interrelated factors will be essential for informing clinical interventions and public health strategies aimed at supporting women with anxiety and depression who turn to cannabis as an alternative treatment.

## Author Contributions

**Jocelyn Mueller:** conceptualization, methodology, formal analysis, investigation, writing – original draft, supervision, software. **Jamia Sapp:** conceptualization, methodology, software, data curation, investigation. **Jennifer Attonito:** conceptualization, methodology, software, data curation, supervision, investigation. **Karina Villalba:** methodology, software, data curation, supervision, investigation. **Aditya Chakraborty:** methodology, writing – review and editing, supervision, investigation.

## Ethics Statement

This study was approved by the Institutional Review Board at the University of Central Florida (Protocol #: IRB00001138, IRB00012110). Informed consent was obtained from all participants being included in the study.

## Conflicts of Interest

The authors declare no conflicts of interest.

## Transparency Statement

The lead author, Jocelyn Mueller, affirms that this manuscript is an honest, accurate, and transparent account of the study being reported; that no important aspects of the study have been omitted; and that any discrepancies from the study as planned (and, if relevant, registered) have been explained.

## Data Availability

The data that support the findings of this study are available from the corresponding author upon reasonable request.
